# The Effect of Distance on Sentence Processing by Older Adults

**DOI:** 10.3389/fpsyg.2019.02455

**Published:** 2019-11-13

**Authors:** Xinmiao Liu, Wenbin Wang

**Affiliations:** ^1^School of English for Specific Purposes, Beijing Foreign Studies University, Beijing, China; ^2^National Research Centre for Foreign Language Education, Beijing Foreign Studies University, Beijing, China

**Keywords:** distance, sentence processing, older adult, aging, sentence comprehension

## Abstract

In sentences with long-distance dependency relations (“*The man whom the police arrested is thin*”), there are two kinds of distance between the gap (object position of *arrested*) and the filler *man*: linear (the intervening words in linear order), and structural (the intervening nodes in the syntactic tree). Previous studies found that older adults have difficulty comprehending sentences with long-distance dependency relations. However, it is not clear whether they are more disrupted by longer structural distance between gaps and fillers, or longer linear distance. There is a distinction between linear distance and structural distance, in that the former is directly related to working memory whereas the latter is associated with syntactic ability. By examining the effect of linear distance and structural distance on sentence processing by older adults, we can identify whether age-related decline in sentence comprehension is attributed to working memory dysfunction or syntactic decline. For this purpose, structural distance and linear distance were manipulated in Mandarin relative clauses (RCs). 30 older adults and 33 younger adults were instructed to perform a self-paced reading task. We found that both groups performed more slowly as structural distance increased, and less accurately when linear distance increased. More importantly, there was a significant interaction between linear distance and age group in the accuracy of comprehension, with linear distance disrupting older adults more than younger adults in offline processing. The findings suggest that the age-related decline in offline sentence comprehension might be attributable to the decline in working memory, rather than syntactic ability. Practical implications, limitations, and directions for future studies are discussed.

## Introduction

Normally aging adults have difficulties comprehending sentences with long-distance dependency relations (LDDs), such as relative clauses (RCs) (Davis and Ball, 1989; Obler et al., 1991). LDDs frequently appear in sentences involving *wh*-movement (“*He helped the man whom the wolf attacked*”), where the filler (*man*) moves from its original syntactic position, and leaves behind it an empty position known as the gap. The comprehension of such sentences requires the processing of syntactic structures. For instance, in processing that sentence “*He helped the man whom the wolf attacked*”, listeners or readers must understand that *man* is the patient of the action *attack* and *wolf* is the agent. They also need to temporarily store the filler *man* in memory in order to integrate it with the gap. Therefore, working memory also plays an essential role in this process. The processing of sentences with LDDs requires both efficient syntactic processing and sufficient working memory resources. A deficiency in either of these two processes can lead to comprehension difficulty. In spite of the numerous studies of the decline of sentence comprehension in older adults (e.g., Kemper, 1986; Zurif et al., 1995; Stine-Morrow et al., 2000; Waters and Caplan, 2001), it is still not clear whether age-related decline in sentence comprehension is the result of syntactic decline or working memory decline.

The study of the distance effect on sentence processing by older adults provides us with a unique opportunity to explore the source of sentence comprehension decline in older adults. For sentences with LDDs, the distance between gaps and fillers can be viewed in two ways, namely the linear way (the intervening words in linear order), and the structural way (the intervening nodes in the syntactic tree) (Hsu and Chen, 2013; Pozniak et al., 2017). There is a distinction between linear distance and structural distance in the sense that the former is directly related to working memory, whereas the latter is related to syntactic processing (Baumann, 2014; Bulut et al., 2018). The memory-based accounts of sentence processing defined distance as the linear distance between the fillers and the gaps, and quantified distance as the number of words intervening between fillers and gaps in the linear order (Gibson, 1998, 2000). As the fillers need to be stored in memory before the gaps are reached, sentences with a longer linear distance require more working memory resources to process. Therefore, linear distance has been regarded as a measure of working memory load in previous studies. If older adults are found to be more affected by the linear distance between gaps and fillers than younger adults, this would indicate that their comprehension difficulty is largely attributed to working memory decline.

On the other hand, structure-based accounts (Collins, 1994; Hamilton, 1995; O’Grady, 1997; Hawkins, 1999, 2004; O’Grady et al., 2003) measured processing difficulty in terms of the structural distance between gaps and fillers, and quantified distance as the number of syntactic nodes between fillers and gaps in the syntactic trees. Structural distance (also referred to as linguistic distance) has been perceived as a measure of linguistic cognitive ability which is largely independent from general cognitive abilities such as working memory (Newmeyer, 1992; Chomsky, 2013b). According to the Principle of Minimal Structural Distance (Chomsky, 2013a, b, 2016), the interpretation of linguistic structures is a structure-dependent process. In understanding or interpreting the meaning of a sentence, one tends to rely more on structural distance or the proximity of words in a hierarchical syntactic tree. If the gap is located further away from the filler in the syntactic tree, it will result in greater difficulty in constructing a syntactic representation of the sentence. Therefore processing difficulty will increase. Structural distance is a typical syntactic property in universal grammar (Chomsky, 2013b), and the comprehension of sentences with longer structural distance usually involves more complex syntactic operations. The difficulty in processing such sentences reflects a deficiency in syntax, rather than working memory. Therefore, if older adults are more disrupted by the longer structural distance between fillers and gaps than younger adults, this would indicate that syntactic decline is a source of age-related decline in sentence comprehension.

Review of the previous accounts of sentence processing indicates that linear distance is mainly a measure of processing difficulty caused by working memory overload, whereas structural distance is largely a measure of syntactic processing difficulty. A study of the effect of the two distance factors on sentence processing may allow us to make inferences about the nature of the decline in sentence comprehension in older adults: that is, whether the age-related decline in sentence comprehension is a result of working memory decline or syntactic decline.

To account for whether language difficulties stem from syntactic impairment or memory limitations, Leivada et al. (2017) proposed the locus preservation hypothesis (LPH), according to which syntax is a resilient part of the human language faculty which is invariably well preserved in patients with language impairment. Syntactic operations are not penetrable to variation across pathologies or languages (Leivada, 2015). The difficulty in language comprehension can be traced back to a brain syntax network (Benítez-Burraco and Murphy, 2016). As syntax evolved early in phylogenetic terms, it results in more robust compensatory mechanisms which make syntax immune and impenetrable to natural or pathological aging as well as developmental disorders. In other words, syntax relies on the brain network that is less novel in evolutionary terms, and thus is more resilient. Cognitive abilities such as working memory, on the other hand, are implemented through a network which is less resilient because it evolved more recently, and its compensatory mechanisms are not yet well established (Leivada et al., 2017). Although the LPH was first proposed to account for language impairment in developmental disorders, it might also be applicable to age-related decline in language processing in aging populations. The LPH explains the better preservation of syntax in phylogenetic terms, and as such it is not narrowly confined to some languages or phenotypes. In other words, the LPH might be universally applicable to both normally aging adults and patients with developmental disorders. The LPH predicts that syntactic ability is well preserved in older adults and the decline in sentence comprehension is largely attributed to cognitive decline such as working memory dysfunction. However, empirical studies on sentence processing in older adults have produced mixed results. Some studies attributed sentence comprehension decline to working memory decline (Kemtes and Kemper, 1997; Caplan and Waters, 2005), whereas others argued for syntax-specific decline as the contributing factor to comprehension difficulty (Zhu et al., 2018; Poulisse et al., 2019). It is still not clear whether the age-related decline in sentence comprehension is the result of working memory dysfunction or syntactic decline. Therefore, whether the LPH can account for age-related decline in sentence comprehension is also still far from clear. The study of the effect of distance on sentence processing by older adults can reveal the possible reasons for age-related decline in sentence comprehension and thus test the validity of the LPH.

Although numerous studies have explored the effect of distance on sentence processing in younger adults (e.g., Hsiao and Gibson, 2003; Lin and Bever, 2006, 2007; Chen et al., 2008; Packard et al., 2011), how distance might influence sentence processing by older adults has remained largely unexplored. So far there has been only one study which directly addressed the effect of distance on the performance of older adults in sentence processing. Zurif et al. (1995) examined the effect of linear distance on English sentence processing by older adults, and found that older adults were more affected by linearly long sentences. However, as there were no younger controls in their study, it was difficult to conclude that the distance effect was related to aging. So far there has been a lack of attempts to examine how structural distance and linear distance might differentially influence sentence processing by older adults. In this study, we intended to explore how the two types of distance might differentially affect sentence processing in older adults. The study provides a fine-grained analysis on the role of syntactic distance in language comprehension by older adults. The findings from the present research enable us to identify whether the decline in sentence processing by older adults is structural-specific or cognitive-specific, and thus further our understanding of the nature of age-related decline in language comprehension.

## Sentence Processing and Distance

Distance is the degree or amount of separation between two linguistic units with syntactic relationships in a sentence. Long-distance dependencies (LDDs) are the dependent relationships between two linguistic units which are linearly apart from each other (Hsu and Chen, 2013). They are also called filler-gap dependencies because they frequently appear in sentences involving *wh*-movement, such as RCs where an element (the *filler*) moves from its original syntactic position, leaving behind it an empty position (the *gap*).

In the relative clause “*He helped the man*_(i)_ *whom the wolf attacked*_*t(i)*_,” the filler is the head noun (“*man*”), and the gap (specified as _*t(i)*_) is the empty position inside the clause modifying the head noun. The head noun *man* is moved from its original syntactic position following *attacked* to the surface position where it is co-indexed with the gap, which allows *man* to gain the patient role of *attacked*. To understand the sentence, the parser needs to resolve the filler-gap dependency relationship by associating the gap with the head noun. Relative clause is one of the most frequently used structures in the investigation into the processing of sentences with LDDs. The studies on RC processing consistently found that subject relative clauses (SRCs) like (1a) are easier to process than object relative clauses (ORCs) like (1b) in various languages such as English (Ford, 1983; Traxler et al., 2002; Gibson et al., 2005; Grodner and Gibson, 2005), Dutch (Mak et al., 2002), German (Mecklinger et al., 1995), or French (Frauenfelder et al., 1980; Holmes and O’Regan, 1981).

(1a) The worker_(i)_ [who _*t(i)*_ sued the driver] regretted it (SRC).
(1b) The worker_(i)_ [whom the driver sued _*t(i)*_] regretted it (ORC).

Previous studies have proposed various accounts to explain this asymmetry in the processing difficulty of RCs. These theories can be summarized in three groups (Pozniak et al., 2017; Bulut et al., 2018), namely the memory-based accounts (Gibson, 1998, 2000), the structure-based accounts (O’Grady, 1997; Hawkins, 1999, 2004), and the frequency-based accounts (Hale, 2001, 2003; Levy, 2008)^1^. Both memory-based accounts and structure-based accounts attributed the asymmetrical processing difficulty to the distance between fillers and gaps, and predicted that longer distance leads to increased processing costs or difficulties in RCs, but they differ in the definition of distance.

The memory-based accounts, as represented by the dependency locality theory proposed by Gibson (1998, 2000), defined the filler-gap distance as the linear distance between head nouns and gaps, and quantified linear distance by the number of words intervening between gaps and fillers in a linear order (Gibson, 1998, 2000). In the memory-based accounts, sentence processing is constrained by working memory capacity. Processing difficulty is evaluated in terms of memory activation at the fillers and the decay in working memory storage before the gaps are reached. For example, for filler-gap integration in English RCs, longer linear distance between gaps and fillers requires more working memory resources and thus, is more difficult to process. Accordingly, English SRCs are easier to process than ORCs because the former are linearly shorter and cognitively less costly than the latter. This account is known as the linear distance hypothesis. Linear distance has been favored mostly by cognitive scientists, and this concept represents the tendency to view distance as a measure of general cognitive abilities, especially working memory. Given the decline of working memory in older adults (Salthouse, 1991; Salthouse and Babcock, 1991; Carpenter et al., 1994), they tend to be more disrupted by the resource demand of texts than younger adults, and thus they might be more sensitive to the manipulation of linear distance (Bopp and Verhaeghen, 2005).

**FIGURE 1:**
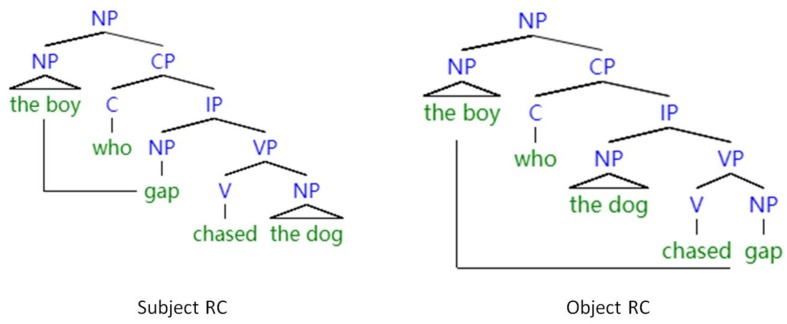
A tree representation of linear vs. structural distance of English RCs.

Structure-based accounts (Collins, 1994; Hamilton, 1995; O’Grady, 1997; Hawkins, 1999, 2004; O’Grady et al., 2003), on the other hand, were concerned with the structural distance between fillers and gaps, and they quantified distance in terms of the crossing nodes between fillers and gaps in syntactic trees (i.e., how deeply the gap is embedded in the tree structure). Structural distance is a concept proposed on the basis of Chomsky’s (1957) transformational generative grammar, which maintains that there are universal underlying syntactic structures across different languages. Accordingly, these accounts propose that processing dynamics are universal in sentence processing. Figure 1 presents a tree diagram of English RCs with the horizontal line representing linear distance and the vertical representing structural distance.

As shown in Figure 1, the gap in the SRC is embedded in the inflection phrase (IP) and the gap in the ORC is within the verb phrase (VP), which is deeper than IP in the tree. This hierarchical relationship in the sentences is assumed by nearly every syntactic theory (O’Grady et al., 2003). There are more intervening syntactic nodes between gaps and fillers in ORCs than in SRCs, which holds true in both head-final languages such as Chinese and head-initial languages such as English or French (Carreiras et al., 2010). For all languages, the structure-based accounts predict that SRCs are easier to process than ORCs. This account is known as the structural distance hypothesis. Structural distance has been regarded as a syntactic parameter (Chomsky, 2013b), and used mostly by theoretical linguists to explore the syntactic features of different languages. Sentences with longer structural distance typically require more complex syntactic operations. The difficulty in processing structurally long sentences reflects a decline in syntactic ability, rather than memory limitations. Therefore, if older adults have greater difficulty in comprehending structurally long sentences, it might provide evidence that a deficiency in syntax underlies their difficulty in sentence comprehension.

As both the structural distance hypothesis and the linear distance hypothesis can explain the asymmetrical processing difficulties between SRCs and ORCs in head-initial languages, the studies on these head-initial RCs cannot disentangle linear distance effect from structural distance effect. For example, as shown in Figure 1, English ORCs are both linearly and structurally longer than SRCs. Therefore, the studies of RC comprehension in head-final languages by older adults cannot reveal whether the decline in sentence comprehension is the result of working memory decline or syntactic decline. As almost all previous studies on sentence processing by older adults focused on head-initial RCs (Zurif et al., 1995; Waters and Caplan, 1996, 1997, 2001; Caplan and Waters, 1999, 2005; Caplan et al., 2011), we are not clear whether older adults’ performance was more affected by structural distance or linear distance, as the two types of distance correlate positively in head-initial RCs.

**FIGURE 2:**
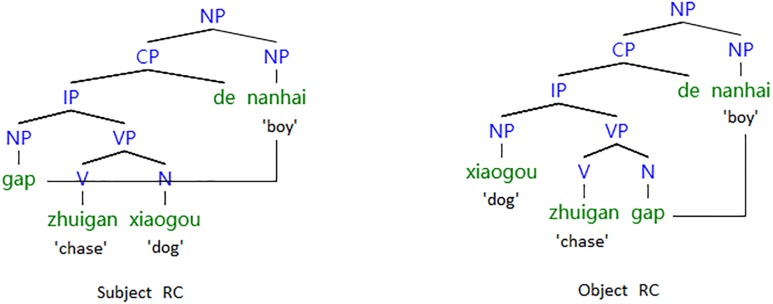
A tree representation of linear vs. structural distance of Chinese RCs.

The research into the differential effect of linear and structural distance has mostly examined head-final RCs, such as Mandarin (Lin et al., 2005; Chen et al., 2008; Packard et al., 2011; Gibson and Wu, 2013), Korean (Kwon et al., 2006; Lee and Stromswold, 2007; Kwon, 2008) and Japanese (Miyamoto and Nakamura, 2003), as the structural distance hypothesis and the linear distance hypothesis made contrastive predictions regarding the processing difficulty. Figure 2 offers a graphical illustration of structural distance and linear distance in Mandarin RCs. As shown in the tree diagram, the linear distance of SRCs is longer than that of ORCs, whereas the structural distance of SRCs is shorter than that of ORCs. Therefore, the linear distance hypothesis predicts that Mandarin SRCs are more difficult than ORCs to comprehend, whereas, according to the structural distance hypothesis, Mandarin SRCs are less difficult than ORCs to process.

The study of head-final RCs can help us to differentiate the effect of linear distance from the effect of structural distance and to find out which type of distance is the major contributing factor to the processing difficulty in older adults. However, previous research into the head-final RCs has largely focused on younger adults (Hsiao and Gibson, 2003; Ishizuka et al., 2003, 2006; Kwon et al., 2006, 2010; Lin and Bever, 2006, 2007; Hsu and Chen, 2007, 2009, 2013; Lee and Stromswold, 2007; Chen et al., 2008; Ueno and Garnsey, 2008; Packard et al., 2011), and little attention has been given to sentence processing by older adults. Zurif et al. (1995) used the lexical priming paradigm to investigate whether older adults were able to construct syntactically governed dependency relations in online sentence processing. In Experiment 1, they found that elderly subjects could fill gaps for SRCs but not for ORCs. In Experiment 2, Zurif et al. (1995) manipulated the linear distance of ORCs and discovered that there was reliable priming at the gaps when there were only five words intervening between antecedents and gaps in ORCs. Zurif et al. (1995) concluded that elderly subjects’ performance was more affected by the linear distance separating gaps and fillers than by the nature of the syntactic representation that had to be constructed. However, as there were no younger controls in their study, it was difficult to conclude that the distance effect resulted from aging. The results of their study are rather difficult to interpret (Caplan and Waters, 1999). Besides, as the lexical priming task used in this study can only reveal whether the filler was activated at the gap position, this study cannot provide enough information regarding the time course of distance effect in sentence processing. Most importantly, in this study as well as other research on sentence processing by older adults (Waters and Caplan, 1996, 1997, 2001; Caplan and Waters, 2005; Caplan et al., 2011), syntactic complexity was measured in terms of linear distance, but structural distance has largely been ignored. The basic argument is that older adults encounter greater difficulty in processing sentences with longer linear distance, as fillers need to be held in working memory before gaps are identified by the parser (Zurif et al., 1995; Grossman et al., 2002). However, there has been a lack of attempts to examine how structural distance and linear distance might differentially influence sentence processing by older adults. Therefore, the question remains whether the age-related decline in sentence processing is driven by the number of intervening words in linear order or the structural complexity of the intervening materials. This study was designed to empirically investigate how linear distance and structural distance influence the age-related decline in sentence processing.

## Materials and Methods

### Design

The key question addressed in this study is whether, and if so, how linear distance and structural distance would affect sentence processing in older adults. Therefore, instead of following the ORC-SRC dichotomy frequently adopted by prior research, we used only SRCs as experimental materials and manipulated the linear distance (long and short) and structural distance (long and short). Using only one type of RCs enabled us to circumvent the confounding factors such as frequency or word order.

The experiment adopted a 2 (linear distance: long and short) × 2 (structural distance: long and short) × 2 (age: old and young) three-factor design. Linear distance and structural distance are within-subjects variables, and age is a between-subjects variable.

### Participants

Thirty older adults (16 female, mean age = 62.83, *SD* = 3.17, age range: 60–73) and 33 younger adults (19 female, mean age = 19.69, *SD* = 2.20, age range: 18–26) from the local community were recruited. All participants were native speakers of Mandarin Chinese. None of the participants reported any history of neurological disease or reading disability. The Chinese version of the Mini-Mental State Examination (Katzman et al., 1988) was implemented among all older adults to screen them for cognitive health before the experiment, and all participants scored over 26 points, indicating all of them were cognitively healthy. The two age groups did not differ significantly in gender ratio, χ^2^ = 0.40, *df* = 1, *p* = 0.526, and years of education, *t*(61) = 0.892, *p* = 0.376.

Using Daneman and Carpenter (1980)’s experimental paradigm, we administered a verbal working memory test. Participants were required to read blocks of sentences, answer comprehension questions, and recall the last words of the sentences. The number of sentences in each block ranged from two to seven. The total number of words recalled correctly was calculated, and served as the final score. The results showed that younger adults (mean = 23.0, *SD* = 2.77) had a larger working memory span than older adults (mean = 20.4, *SD* = 2.91), *t*(61) = 3.62, *p* < 0.05.

The experiment was implemented in accordance with the recommendations of the Ethics Committee of Beijing Foreign Studies University. The protocol was approved by the Ethics Committee of Beijing Foreign Studies University. All subjects in the experiment provided written informed consent in accordance with the Declaration of Helsinki, and received monetary compensation for their engagement at the end of the experiment.

### Experimental Stimuli

We followed Hsu and Chen (2013)’s study to adopt pseudo-cleft sentences to avoid the garden-path interpretation caused by the lack of left boundary of Chinese RCs. Pseudo-cleft sentences are the structures containing free RCs as sentential subjects and noun phrases as sentential objects. As exemplified in Table 1, the sentential subject, *guke xuyao de* (“what customers want”), is a free RC, and the sentential object, *younaixin de fuwuyuan* (“waiters who have patience”), is a noun phrase modified by a RC. This structure can bias the parser for the correct expectation of a RC.

**TABLE 1:** Sample pseudo-cleft sentence.

	**Subject**	**Verb**	**Object**
Experimental sentence	guke xuyao de	shi	younaixin de fuwuyuan
Glossed sentence	customers need de	are	have patience de waiter
English equivalence	“What customers need are the waiters who have patience.”		

**FIGURE 3:**
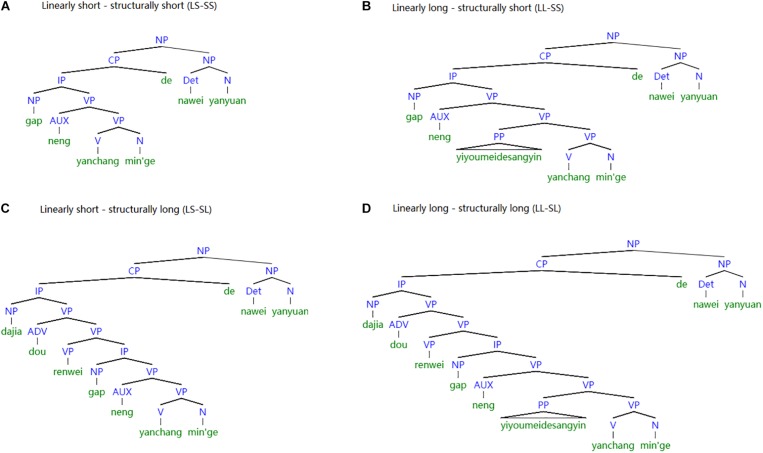
Structural representation of the sample experimental stimuli. **(A)** Linearly short – structurally short (LS-SS). **(B)** Linearly long – structurally short (LL-SS). **(C)** Linearly short – structurally long (LS-SL). **(D)** Linearly long – structurally long (LL-SL).

Linear distance (short and long) and structural distance (short and long) were manipulated, resulting in four types of sentences, namely: RCs with short linear distance and short structural distance (LS-SS); RCs with long linear distance and short structural distance (LL-SS); RCs with short linear distance and long structural distance (LS-SL); and RCs with long linear distance and long structural distance (LL-SL). The sentences consist of three parts: a context sentence to start the trial, a target sentence with RCs, and a phrase to end the trial. An example sentence was provided in Table 2 (see Supplementary Appendix S1 for a complete experimental sentence). The tree representation of the experimental sentences can be found in Figure 3.

**TABLE 2:** Sample experimental sentences.

**Type**	**Sample sentence**
Linearly short, Structurally short (LS-SS)	1. Context (Region 1-9) 2. … *guanzhong*_10_ *xihuande*_1__1_ *shi*_12_ [*neng*_13_ *yanchang*_14_ *min’ge*_15_ *de*_16_] *nawei*_17_ *yanyuan*_18_‘The actor the audience liked was the one who could sing folksongs.’ 3. A sentence-final phrase (*bushi lingyiwei* ‘not the other one’) (Region 19)
Linearly long, Structurally short (LL-SS)	1. Context (Region 1-6) 2. … *guanzhong*_7_ *xihuande*_8_ *shi*_9_ [*neng*_10_ *yi*_11_ *youmeide*_12_ *sangyin*_13_ *yanchang*_14_ *min’ge*_15_ *de*_16_] *nawei*_17_ *yanyuan*_1__8_ ‘The actor the audience liked was the one who could sing folksongs using his beautiful voice.’ 3. A sentence-final phrase (*bushi lingyiwei* ‘not the other one’) (Region 19)
Linearly short, Structurally long (LS-SL)	1. Context (Region 1-6) 2. …*guanzhong*_7_ *xihuande*_8_ *shi*_9_ *dajia*_10_ *dou*_11_ *renwei*_12_ [*neng*_13_ *yanchang*_14_ *min’ge*_15_ *de*_16_] *nawei*_17_ *yanyuan*_18_ ‘The actor the audience liked was the one who everyone thought could sing folksongs.’ 3. A sentence-final phrase (*bushi lingyiwei* ‘not the other one’) (Region 19)
Linearly long, Structurally long (LL-SL)	1. Context (Region 1-3) 2. …*guanzhong*_4_ *xihuande*_5_ *shi*_6_ *dajia*_7_ *dou*_8_ *renwei*_9_ [*neng*_10_ *yi*_11_ *youmeide*_12_ *sangyin*_13_ *yanchang*_14_ *min’ge*_15_ *de*_16_] *nawei*_17_ *yanyuan*_1__8_ ‘The actor the audience liked was the one who everyone thought could sing folksongs using his beautiful voice.’ 3. A sentence-final phrase (*bushi lingyiwei* ‘not the other one’) (Region 19)

*LL-SL, Linearly long and structurally long; LS-SL, Linearly short and structurally long; LL-SS, Linearly long and structurally short; LS-SS, Linearly short and structurally short. The numbers in subscript represent the region numbers.*

The critical regions are the RC verb (Region 14), RC object (Region 15), RC marker *de* (Region 16), determiner and classifier (Region 17), and head noun (Region 18). They were matched for lexical items across the four conditions. The context sentences at the beginning of each trial were customized for each condition to ensure the total length of the sentences was the same. The context sentences introduced the two agents which served as the head nouns for the upcoming RCs. This arrangement ensured that participants would have the expectation of a RC, rather than a main clause (Crain and Steedman, 1985; Altmann, 1989; van Berkum et al., 1999; Grodner et al., 2005). In the RC segments, there were four words intervening between gaps and fillers in linearly short sentences (e.g., *neng yanchang min’ge de* “can sing folk song DE”), and in linearly long sentences a three-word adverbial phrase was added, resulting in a seven-word interval between gaps and fillers (e.g., *neng yi youmei-de sangyin yanchang min’ge de* “can use beautiful voice sing folk song DE”). In the structurally long sentence, RC was introduced by a complement clause which contained a subject noun (*dajia* “everyone”), an adverb (*dou* “all”), and a verb (*renwei* “think”). No complement clauses were embedded in structurally short sentences. A phrase (Region 19) was added to the last segment of the sentence to differentiate the wrap-up effect from the distance effect.

In this study, we devised 24 sets of experimental sentences and generated four lists using the Latin-square design, including 24 sentences in each list (six in each condition). 72 fillers were added which were irrelevant to the aim of the experiment. Both experimental sentences and fillers were followed by comprehension questions which asked about different regions of the sentences to ensure that subjects focused equally on all sentential segments. All the comprehension questions were yes/no questions, and thus the structure of the questions did not differ between different experimental conditions, which ensured that the accuracy was based on the comprehension of experimental items, rather than on the understanding of the questions. The questions were presented in their entirety at the center of the computer screens and remained there until participants indicated their responses. Half of the comprehension questions had an expected answer of YES, and the other half had an expected answer of NO. The comprehension questions were used to assess participants’ offline reading performance and to check whether they performed the experimental task carefully and properly. The data from the participants who answered over 70% of the comprehension questions correctly were included in the statistical analysis. The 96 experimental sentences were pseudo-randomized before they were presented to participants. To ensure the four conditions matched in plausibility, we administered a plausibility rating test among 40 adults. None of them participated in the experiment. Results of ANOVA revealed no significant difference between the four types of RCs in plausibility rating, *F*(3, 20) = 1.14, *p* = 0.359.

### Procedure

A self-paced reading task was implemented to assess participants’ performance in sentence processing. The experiment was designed with E-Prime 2.0. During the experiment, participants read sentences word by word and pressed the spacebar on the keyboard as soon as they finished reading a word. Then the following word appeared on the screen and replaced the previous word. At the end of the sentences, participants were asked to answer comprehension questions as fast as they could by pressing “1” for YES and “0” for NO. The answers (YES/NO) were counterbalanced across different conditions. Participants completed eight practice questions before the formal experiment. The computers recorded the reading times (RTs) and accuracy.

## Results

### Accuracy

**FIGURE 4:**
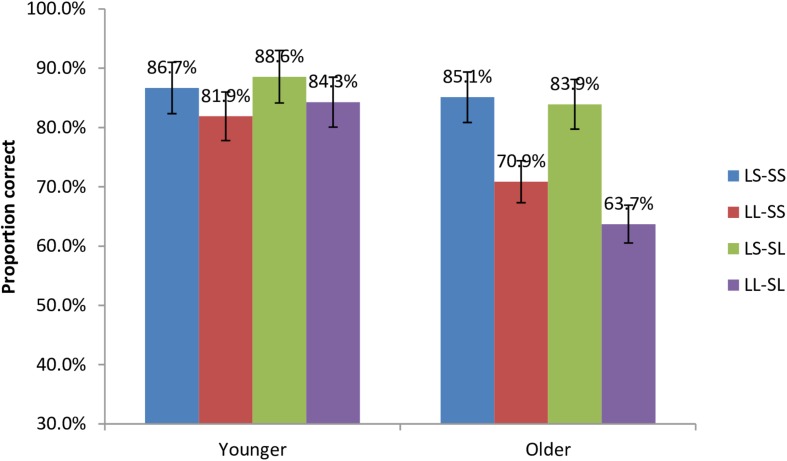
Proportion correct by age group.

Figure 4 presents a graphic summary of mean accuracy rate by age group. The mean accuracy was 82.1% for younger adults and 66.7% for older adults. Although older adults performed significantly worse than younger adults, both groups achieved the above chance level, *p*s < 0.05.

In this study, we used mixed-effects modeling to assess the effect of experimental factors, using the lme4 package (Bates et al., 2014) in R (R Development Core Team, 2014). Logistic mixed-effects regression modeling was used to analyze accuracy data. The model included linear distance (long, short), structural distance (long and short), age group (old and young) and all their interactions as predictors, and subjects and items as random intercepts. Satterthwaite’s approximation was used to estimate *p*-values. Tukey’s HSD tests were performed to make a pairwise comparison. The result of the analysis indicates that there was a significant effect of age group, ß = −1.26, *SE* = 0.026, *z* = −4.92, *p* < 0.001, and a significant interaction between age group and linear distance, ß = 0.86, *SE* = 0.39, *z* = 2.19, *p* < 0.05. Pair-wise comparison shows that older adults performed significantly less accurately than younger adults. In the younger group, the effect of linear distance was not significant (*p* = 0.201), whereas a significant effect of linear distance was found in older adults (*p* < 0.01).

### Reading Times

**FIGURE 5:**
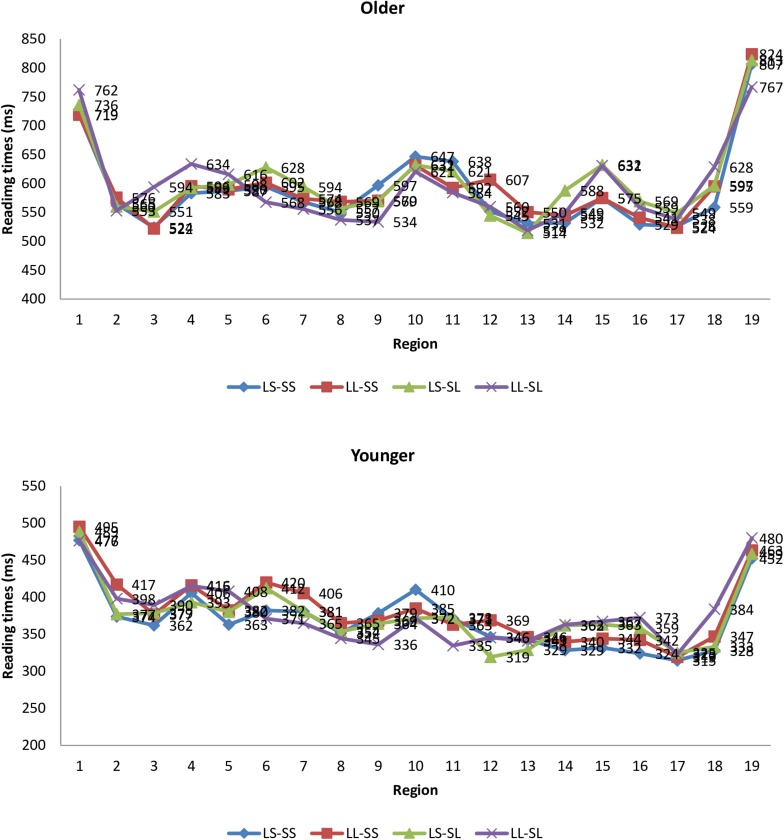
Mean reading times (RTs) by sentence segment and age group.

Reading times under 100 ms and beyond three standard deviations (SDs) from the mean were considered as outliers and removed. These procedures resulted in 1.61% of the data excluded from analysis. The trials which were incorrectly understood (based on performance on comprehension questions) were excluded from the analyses. After data trimming, RTs were analyzed using linear mixed-effects modeling with linear distance, structural distance, age and their interactions as predictors, and subject and item as random intercepts. Tukey *post hoc* tests were applied to explore the significant interactions. The average RTs for each sentential segment are presented in Figure 5.

In the RC verb segment (“*yanchang*”), results of mixed effects modeling revealed that there was a significant effect of age group, ß = 0.41, *SE* = 0.08, *t* = 5.22, *p* < 0.001, and a significant effect of structural distance, ß = −0.08, *SE* = 0.03, *t* = −3.10, *p* < 0.01. Pair-wise comparison shows that older adults were slower than younger adults. The sentences with long structural distance were read more slowly than those with short structural distance. No significant interaction effects were found in the RC verb region (*F*s < 1).

In the RC object (“*minge*”), there was a significant effect of age group, ß = 0.53, *SE* = 0.09, *t* = 6.02, *p* < 0.001, and a significant effect of structural distance, ß = −0.07, *SE* = 0.03, *t* = −2.15, *p* < 0.05. No significant interaction effects were found in this region (*F*s < 1). Younger adults were significantly faster than older adults. The sentences with long structural distance were read more slowly than those with short structural distance.

In the RC marker (“*de*”), statistical analysis revealed a significant effect of age group, ß = 0.41, *SE* = 0.07, *t* = 6.14, *p* < 0.001, and a significant effect of structural distance, ß = −0.08, *SE* = 0.03, *t* = −3.33, *p* < 0.05. No other effects were found to be significant. Pair-wise comparison found that younger adults were significantly faster than older adults. The structurally longer sentences were processed more slowly than shorter sentences.

In the classifier (“*nawei*”), there was a significant effect of age group, ß = 0.49, *SE* = 0.07, *t* = 6.93, *p* < 0.001. RTs for older adults were significantly longer than those for younger adults. No other effect was significant.

In the head noun (“*yanyuan*”), we found a significant effect of age group, ß = 0.49, *SE* = 0.09, *t* = 5.73, *p* < 0.001; a significant effect of linear distance, ß = −0.11, *SE* = 0.03, *t* = −4.31, *p* < 0.001; a significant effect of structural distance, ß = −0.10, *SE* = 0.03, *t* = −3.40, *p* < 0.001; and a significant interaction between linear distance and structural distance, ß = 0.10, *SE* = 0.04, *t* = 2.50, *p* < 0.05. Pair-wise comparison shows that younger adults were faster than older adults. When the linear distance was long, the effect of structural distance was significant, with structurally long sentences read more slowly than structurally short sentences. When linear distance was short, no significant difference was found in RTs between structurally long sentences and structurally short sentences.

To further interpret the main effect of age on the RTs, we also analyzed the RTs at the two pre-critical regions immediately before the critical regions. We discovered a significant effect of age group at both regions, ß = 0.50, *SE* = 0.08, *t* = 6.44, *p* < 0.001; ß = 0.42, *SE* = 0.08, *t* = 5.28, *p* < 0.001. No other significant effect was found in the pre-critical regions (*F*s < 1). The RTs for older adults were consistently longer than those for younger adults. The findings indicate that older adults were significantly slower than younger adults in these two regions.

## Discussion

Many studies show that the ability to process sentences with long distance dependency relations declines with advancing age (e.g., Davis and Ball, 1989; Obler et al., 1991). However, it is still not clear whether the age-related decline in sentence processing is the result of working memory decline or syntactic decline. To explore the potential source of the decline in sentence processing, the current research examined how linear distance and structural distance contributed to the age-related change in the processing of Mandarin head-final RCs, using a self-paced reading paradigm. The findings indicate that older adults performed significantly more slowly and less accurately than younger adults. Both the older and younger groups performed less efficiently in processing structurally longer sentences in online reading, and less accurately in comprehending linearly longer sentences at the offline stage. More importantly, a significant interaction between linear distance and age group was found in the accuracy of comprehension. Linear distance disrupted older adults more than younger adults at the offline stage, which suggests that there is an age-related decline in comprehending linearly long sentences at the offline stage. The finding provided evidence that working memory decline is a crucial source of age-related decline in offline sentence comprehension, a finding which supports the predictions of the LPH.

The findings about the effect of linear distance are consistent with Zurif et al.’s (1995) study that also found a significant effect of linear distance on sentence processing among older adults. However, as Zurif et al. (1995) did not include younger controls, it was not clear whether the effects were related to aging. Our findings showed the age difference in the effect of linear distance was only shown at the offline stage. In online processes, although the effect of linear distance was significant at head nouns in both older and younger adults, there was no significant age difference, which suggests that the effect of linear distance on online processing did not change with advancing age. The findings that the effect of linear distance was only found with head nouns were inconsistent with previous studies on head-initial RCs which found a significant effect of linear distance throughout the sentences (e.g., Crain and Fodor, 1985; Stowe, 1986; Frazier, 1987; Frazier and Clifton, 1989; Frazier and d’Arcais, 1989). These studies supported the Active Filler Strategy theory, according to which the parser will actively search for a gap to reduce the cognitive burden as soon as the filler is identified (Frazier, 1987). The inconsistency might be caused by the structural differences between Chinese and English RCs. As Chinese RCs are head-final structures, RCs come before head nouns and as a result, gaps precede fillers, which might reduce the memory cost as the gaps are semantically empty (Hsu and Chen, 2013). The storage cost may be relatively low compared with English RCs. The fact that there was a significant effect of linear distance at head nouns might be due to the increased integration costs, as it is at the head nouns that the parser starts to associate the fillers with the gaps. This finding was consistent with Hsu and Chen (2013)’s eye tracking study which also discovered a delayed effect of linear distance in Chinese RC processing.

The greater sensitivity to linear distance in offline processing among older adults might be attributed to the insufficiency of working memory resources. As linear distance is a variable closely associated with the working memory burden of the linguistic structures being processed, an increase in linear distance would increase the processing effort and tax working memory resources (Gibson, 1998, 2000). Due to the decline in working memory capacity in older adults (Salthouse, 1991; Carpenter et al., 1994), they might have greater difficulty in processing linearly long sentences. Since younger adults have relatively sufficient cognitive resources available, they tend to be less disrupted by linear distance compared with older adults. The age difference in the effect of linear distance suggests that working memory is one of the key contributing factors to the age-related decline in sentence comprehension, an argument supported by many prior studies (Kemper, 1986, 1987; Baum, 1993; Waters and Caplan, 1996, 1997, 2001; Burke and MacKay, 1997).

Interestingly, this study revealed a discrepancy in the effect of linear distance between online and offline measures. Although the effect of linear distance did not differ between older and younger adults in online sentence processing, it was stronger in older adults at the offline stage than in younger adults. This discrepancy might be attributed to the different cognitive mechanisms underlying online and offline sentence processing. Caplan and Waters (1999) distinguished between interpretive processing (online) and post-interpretive processing (offline), with the former referring to the extraction of meaning from linguistic signals, and the latter referring to the use of the propositional content to perform tasks such as reasoning or storing information in long-term memory. According to Caplan and Waters (1999)’s separate verbal working memory theory, the working memory resources recruited by online or interpretive processing are independent from the general working memory resources called on by offline processing. The working memory measured by traditional assessment tools, such as the Daneman and Carpenter task, merely constrains the offline processes, but not the online processes. Online sentence processing is an automatic and unconscious process which is self-sufficient in terms of memory consumption. As one of the most over-practiced skills that human beings engage in Caplan et al. (2013), online sentence processing is immune to both natural aging, and pathological aging such as Alzheimer’s disease (Waters and Caplan, 1997). As Caplan and Waters (1999) have pointed out, most of the tasks in which older adults have greater difficulty than younger adults are those that require post-interpretive or offline processing, such as retaining, recalling or re-ordering a large amount of information in memory (Kemper, 1986; Light, 1990), or comprehending implausible sentences (Davis and Ball, 1989; Obler et al., 1991). In the present study, the online reading process is an interpretive process and the sentence-final questions are actually a post-interpretive process during which participants used the meaning they had extracted from online reading processing to answer the comprehension questions. Older adults were more affected by linearly long sentences than younger adults only in offline measures, due to their decline in general working memory. As online processing is relatively less affected by aging and the related decline in working memory, this might explain why no significant age difference in the effect of linear distance has been found in the online measures.

This study also found that both older adults and younger adults had greater difficulty comprehending structurally long sentences than structurally short sentences. However, no significant interaction between age and structural distance was found in either online or offline processes, which suggests that the age difference was not significant in the effect of structural distance. The sensitivity to structural distance might be relatively stable across the life span from youth to old age. The findings may reveal the important underlying mechanisms for language in the aging brain. As we have mentioned earlier, linear distance and structural distance are not simply syntactic or structural parameters. Rather they represent the underlying cognitive ability which plays a crucial role in language processing. Linear distance is considered as a measure of general cognitive ability, whereas structural distance reflects linguistic cognitive ability. The finding that adults in old age were particularly affected by linear distance might indicate that the age-related decline in sentence comprehension is more relevant to the decline in general cognitive abilities (e.g., working memory). Given the lack of significant age-related differences in the effect of structural distance on sentence processing, syntactic operations *per se* might be resilient to aging. This finding is supported by many studies on the underlying neural mechanisms of syntactic processing among older adults, which found that syntactic knowledge and skills were relatively well preserved in normally aging adults (Shafto and Tyler, 2014; Campbell et al., 2016; Samu et al., 2017), and is also consistent with the prediction of the LPH.

In online processes, we found no significant age difference in the effect of either linear or structural distance. The only significant age-related difference is that older adults were generally slower than younger adults at both the critical regions and pre-critical regions. This is consistent with most previous studies on sentence processing by aging adults (Caplan and Waters, 2005; Caplan et al., 2011; Liu et al., 2019). This finding is supported by Salthouse (1996)’s Processing Speed Theory of Adult Age Differences, according to which the increase in age is typically connected to the reduction in processing speed with which many cognitive operations are executed. This decrease in processing speed can impair many cognitive activities including sentence processing. Apart from the age-related general slowing, another contributing factor is the lower familiarity with computer-based procedures in older adults. As younger adults are typically more experienced and skilled at operating computers (Charness et al., 2010; Kosowicz and MacPherson, 2016), they might be faster to push buttons than older adults. Therefore, we need to be cautious in interpreting the experimental results regarding the age differences in the speed of performance. The slower speed of performance in older adults might be attributed to either age-related general slowing or lower familiarity with computer-based procedures in older adults.

A possible reason for the lack of significant age difference in the effect of distance is related to the age factor. What needs to be noticed is that most older participants in our study were in their early 60s and only one was over 70. According to Forman et al. (1992)’s sub-group definition, most of these participants should be defined as “young-old” (60–69 years old), which suggests that they are relatively younger in the entire aging population. Davis and Ball (1989) reported that reading difficulty was found only in participants in their 70s. A study with Chinese-speaking older adults (Liu, 2015) found that 70 years old is the critical turning point for the decline in language ability in Chinese aging population. In Liu (2015)’s study, significant decline in language ability was only witnessed in the subjects over 70 years old. As most of our participants were under 70 years old, this might be the reason why we did not find significant age difference in the effect of linear or structural distance. The findings from our study mainly suggest that online sentence processing was relatively well preserved in the older adults in their early 60s. However, as this study only investigated the older adults in early 60s, it is difficult to conclude that online sentence processing remains completely intact with aging. Further efforts are needed to extend this study to older adults with more advancing age in order to provide a more comprehensive picture about whether, and if so, how the ability to process sentences changes in aging populations.

The findings from the current study have important practical implications for the identification of age-related impairments in language comprehension. Studies of language processing by normally aging adults can provide important baseline information for medical professionals to diagnose and evaluate language impairments in pathologically aging adults, such as patients with Alzheimer’s disease. As this study has revealed a significant decline in the comprehension of linearly long sentences, linear distance can serve as a useful parameter for the assessment of age-related decline in language comprehension. However, given that this study did not cover a large sample, more extensive research is needed before its application in medical practice. Another major limitation is that we tested only a small number of experimental sentences, which might limit the power of this research. Future studies might replicate this study among a larger sample, and with a larger number of items, to better understand the effect of distance on sentence processing by older adults. Besides, the comparison between healthy aging adults and AD patients can provide important information for the diagnosis and evaluation of language impairments in AD. Future studies are needed to explore how the two types of older adults differ in sentence comprehension, and how syntactic parameters such as linear or structural distance might influence their performance.

## Conclusion

This study investigated the influence of linear distance and structural distance on the processing of sentences with LDDs by older adults, using a self-paced reading task. The results indicated that older adults performed more slowly and less accurately than younger adults. Both age groups processed structurally long sentences less efficiently in online processing and comprehended linearly long sentences less accurately in offline processes. Compared with younger adults, there was a stronger effect of linear distance on the offline performance of older adults. Older adults were more disrupted by the longer linear distance between gaps and fillers than younger adults, which indicates that the age-related decline in offline sentence processing might be more attributed to working memory decline than the impairment in syntactic competence. Additional research is needed to further explore the role of linguistic or cognitive ability in sentence comprehension by older adults, and more structural variables need to be investigated to better understand the age-related changes in sentence comprehension.

## Data Availability Statement

The datasets generated for this study are available on request to the corresponding author.

## Ethics Statement

The studies involving human participants were reviewed and approved by the Ethics Committee of Beijing Foreign Studies University. The participants provided their written informed consent to participate in this study.

## Author Contributions

XL conceived and designed the study, analyzed data of the study, and wrote the manuscript. WW conceived and supervised the research.

## Conflict of Interest

The authors declare that the research was conducted in the absence of any commercial or financial relationships that could be construed as a potential conflict of interest.

## Footnotes

1 The frequency-based accounts provide important explanations for the asymmetrical processing difficulty between SRCs and ORCs. The basic assumption is that the processing dynamics are influenced by the frequency of linguistic structures (Reali and Christiansen, 2007). The more frequent a given linguistic structure is, the easier it is to process. Typical frequency-based theories include the surprisal theory (Hale, 2001; Levy, 2008; Staub, 2010), the entropy reduction theory (Hale, 2003, 2006) and the tuning hypothesis (Mitchell et al., 1995; Jurafsky, 1996). However, as no evidence has been found that these accounts are relevant to sentence comprehension in an aging population, they do not seem to be very relevant to the research question this study intended to address. Therefore, we did not elaborate on these accounts in this paper. Those who are interested can refer to Van Schijndel (2017) for a systematic review.
