# Supplementary material for: The Effect of Distance on Sentence Processing by Older Adults
Source: Front Psychol. 2019 Nov 13;10:2455. doi: 10.3389/fpsyg.2019.02455 (PMC6865351; doi:10.3389/fpsyg.2019.02455)
Supplement: Supplementary file 1 [file Table_1.doc]

**Appendix 1. Example sentences for the experiment**

| **Type** | **Stimuli** |
| --- | --- |
| LS-SS | 根据新的规划和安排，导演选用了两名演员，观众喜欢的是能演唱民歌的那位演员，不是另一位。  ‘According to the new plans and arrangements, the director selected two actors. The actor the audience liked was the one who could sing folksongs, not the other one.’ |
| LL-SS | 据新的安排，导演选用了两名演员，观众喜欢的是能以优美的嗓音演唱民歌的那位演员，不是另一位。  ‘According to the new arrangements, the director selected two actors. The actor the audience liked was the one who could sing folksongs using his beautiful voice, not the other one.’ |
| LS-SL | 根据新的安排，导演选用了两名演员，观众喜欢的是大家都认为能演唱民歌的那位演员，不是另一位。  ‘According to the new arrangements, the director selected two actors. The actor the audience liked was the one who everyone thought could sing folksongs, not the other one.’ |
| LL-SL | 导演选用了两名演员，观众喜欢的是大家都认为能以优美的嗓音演唱民歌的那位演员，不是另一位。  ‘The director selected two actors. The actor the audience liked was the one who everyone thought could sing folksongs using his beautiful voice, not the other one.’ |

LS-SS: Linearly short – structurally short; LL-SS: Linearly long – structurally short; LS-SL: Linearly short – structurally long; LL-SL: Linearly long – structurally long.
